# Long-Term Elevated Inflammatory Protein Levels in Asymptomatic SARS-CoV-2 Infected Individuals

**DOI:** 10.3389/fimmu.2021.709759

**Published:** 2021-09-17

**Authors:** Liina Tserel, Piia Jõgi, Paul Naaber, Julia Maslovskaja, Annika Häling, Ahto Salumets, Eva Zusinaite, Hiie Soeorg, Freddy Lättekivi, Diana Ingerainen, Mari Soots, Karolin Toompere, Katrin Kaarna, Kai Kisand, Irja Lutsar, Pärt Peterson

**Affiliations:** ^1^Molecular Pathology, Institute of Biomedicine and Translational Medicine, University of Tartu, Tartu, Estonia; ^2^Children’s Clinic of Tartu University Hospital, Tartu, Estonia; ^3^Department of Pediatrics, Institute of Clinical Medicine, University of Tartu, Tartu, Estonia; ^4^SYNLAB Estonia, Tallinn, Estonia; ^5^Department of Microbiology, Institute of Biomedicine and Translational Medicine, University of Tartu, Tartu, Estonia; ^6^Institute of Technology, University of Tartu, Tartu, Estonia; ^7^Department of Pathophysiology, Institute of Biomedicine and Translational Medicine, University of Tartu, Tartu, Estonia; ^8^Clinical Research Centre, Tartu University Hospital, Tartu, Estonia; ^9^Family Doctor Center Järveotsa, Tallinn, Estonia; ^10^Family Doctor Center Kuressaare, Kuressaare, Estonia; ^11^Department of Epidemiology and Biostatistics, Institute of Family Medicine and Public Health, University of Tartu, Tartu, Estonia; ^12^Clinical Research Centre, Institute of Clinical Medicine, University of Tartu, Tartu, Estonia

**Keywords:** SARS-CoV-2 infection, antibodies, T cell response, inflammation markers, S100A12, OSM

## Abstract

The clinical features of SARS-CoV-2 infection range from asymptomatic to severe disease with life-threatening complications. Understanding the persistence of immune responses in asymptomatic individuals merit special attention because of their importance in controlling the spread of the infections. We here studied the antibody and T cell responses, and a wide range of inflammation markers, in 56 SARS-CoV-2 antibody-positive individuals, identified by a population screen after the first wave of SARS-CoV-2 infection. These, mostly asymptomatic individuals, were reanalyzed 7-8 months after their infection together with 115 age-matched seronegative controls. We found that 7-8 months after the infection their antibodies to SARS-CoV-2 Nucleocapsid (N) protein declined whereas we found no decrease in the antibodies to Spike receptor-binding domain (S-RBD) when compared to the findings at seropositivity identification. In contrast to antibodies to N protein, the antibodies to S-RBD correlated with the viral neutralization capacity and with CD4^+^ T cell responses as measured by antigen-specific upregulation of CD137 and CD69 markers. Unexpectedly we found the asymptomatic antibody-positive individuals to have increased serum levels of S100A12, TGF-alpha, IL18, and OSM, the markers of activated macrophages-monocytes, suggesting long-term persistent inflammatory effect associated with the viral infection in asymptomatic individuals. Our results support the evidence for the long-term persistence of the inflammation process and the need for post-infection clinical monitoring of SARS-CoV-2 infected asymptomatic individuals.

## Highlights:

- Antibodies to SARS-CoV-2 nucleocapsid decline in asymptomatic individuals 7-8 months after the infection.
- Antibodies to S-RBD correlate with virus neutralization and CD4^+^ T cell responses.
- Asymptomatic individuals have elevated serum levels of S100A12, TGF-alpha, IL18, and OSM protein markers 7-8 months after their infection, suggesting a persistent inflammatory process.

## Introduction

The clinical manifestations of SARS-CoV-2 infection range from asymptomatic disease to severe pneumonia with acute respiratory distress syndrome. Among all infected individuals, the asymptomatic cases form a large group as altogether 25-45% of PCR positive individuals have been reported minimally symptomatic or asymptomatic to COVID-19 (1). Despite mild or no disease manifestation, the role of asymptomatic individuals in SARS-CoV-2 transmission is considered significant (2, 3). Their SARS-CoV-2 viral load is similar to symptomatic patients as determined by PCR tests in nasopharyngeal swabs (1) and they can efficiently transmit the virus, emerging as major spreaders of the virus.

Due to the lack of clinical symptoms, asymptomatic cases are difficult to identify, and there is less longitudinal data on their long-term immune responses. The asymptomatic individuals mount efficient immediate innate immune responses supported by analysis of SARS-CoV-2 antibodies (4, 5) and B cell numbers (6). The main viral antibody targets are the SARS-CoV-2 Nucleocapsid (N) protein and the Spike glycoprotein and its receptor-binding domain (S-RBD), which is the target for neutralizing antibodies and vaccine development. However, the long-term persistence of antibodies in asymptomatic individuals is less known and most studies have reported results within six months after the infection (5, 7–10).

The SARS-CoV-2–specific T cell immune responses in asymptomatic are similar or even more efficient than in symptomatic individuals (11), and they appear to have functionally complete memory T cell responses. In particular, increased activity of effector CD4^+^ T cells in the asymptomatic individuals may be derived from pre-existing T cell immunity to SARS-CoV-2 *via* natural exposure or infection promoting early rapid control of the disease and preventing the pathology seen in severe COVID-19 patients (12, 13). Nevertheless, their antibody levels and T cell responses have been reported lower or declining (5, 9, 13, 14). Furthermore, their immune response has been described, in contrast to severe cases, to have unique immunological features with strong innate immune response and early type 1 immunity with high interferon-gamma production (11, 15–18).

The SARS-CoV-2 severe infection results in acute lung inflammation, which is caused by activated macrophages and monocytes, and T cells, accompanied by activation-induced apoptosis and lymphopenia. Most asymptomatic cases have normal blood laboratory findings but ground-glass opacities in the lungs are present in over half of the cases as revealed by radiology (3). Very few studies have addressed blood inflammation markers during the acute infection or after the viral clearance in asymptomatic cases and patients with mild disease, although the reports on post-acute infectious consequences of COVID-19 are now emerging (19).

To get insight into the persistence of immune response, we measured antibody and T cell responses, and inflammation marker levels in asymptomatic individuals after 7-8 months of their infection. We show in our long-term analysis that asymptomatic patients have efficient neutralizing S-RBD antibody levels and mount virus-specific T cell responses against different structural proteins similar to symptomatic COVID-19 cases. The analysis also revealed unexpected elevated levels of inflammation markers 7-8 months post-infection, suggesting long-term inflammatory effect or increased susceptibility of viral infection of asymptomatic individuals.

## Materials and Methods

### Samples

The Ethical Committee (The Ethical Committee at the University of Tartu) approval (#323T-21) was received for the studies. The participants provided their written informed consent to participate in the study. The SARS-CoV-2 antibody-positive individuals were identified by KoroSero-EST-1 cross-sectional seroepidemiological study conducted from May 8 to July 31, 2020, in two general practitioners at Tallinn and Saaremaa island and is presented in detail elsewhere (20). The population in Saaremaa, in particular, had an outbreak of COVID-19 in March 2020 with a seroprevalence of 6.3%.

The 56 antibody-positive individuals were reanalyzed for the longitudinal samples in November 2020, which occurred on average 7-8 months after their initial infection in March 2020 i.e. 6 months after the seropositivity analysis (**Table 1**). From these, 44 individuals had the asymptomatic infection as they did not report acute respiratory disease or other COVID-19 symptoms since March 2020 but were seropositive for SARS-CoV-2 antibodies. Twelve antibody-positive individuals reported symptoms of respiratory disease. As negative controls, we studied 115 age and sex-matched uninfected individuals from Saaremaa and Tallinn populations. Samples for serum analysis were kept +4°C during the transportation to the laboratory, where stored at -20°C until testing in SYNLAB Estonia Central Laboratory or at the research laboratories of the University of Tartu, Estonia.

**Table 1:** Study participants, their seropositivity status, age, sex and numbers analyzed in each assay.

SARS-CoV-2 status	Number	Age years(mean and range)	Sex F/M	CLIA	LIPS	T cell	Olink
Antibody-positive	56	48.5 (11–101)	38/18	37	56	45	50
*Asymptomatic*	*44*	*47.0* (11–99)	*31/13*	*29*	*44*	*34*	*39*
*Symptomatic*	*12*	*53* (18–101)	*7/5*	*8*	*12*	*11*	*11*
Antibody-negative	115	48.5 (10–85)	79/36	115	115	41	54

### Antibody Testing

The samples were tested by chemiluminescent microparticle immunoassay (CLIA) for detection of IgG against SARS-CoV-2 nucleoprotein (N) (Abbott Architect SARS-CoV-2 IgG with ARCHITECT i2000SR analyzer; Abbott Laboratories, USA) according to the manufacturer’s instructions and as reported earlier (20, 21). Luciferase-based immunoprecipitation assays (LIPS) with S-RBD and N proteins were performed as reported (22). Results are expressed as percent of positive control's luciferase activity values (the discrimination level was 1.6 for S-RBD, and 1.3 for N antigens).

### Neutralization Assay

An in-house neutralization assay was performed for positive samples as reported (20). The neutralization assay was done in duplicates, the serum samples were two-fold serially diluted in Virus Growth Media (VGM – DMEM/0.2% BSA/Pen-Strep) starting from 1:4 to 1:4096 in a volume of 50 μl per well. The viral stock was diluted in VGM to contain 100 pfu (plaque-forming units) per 50 μl. Virus (a local Estonian SARS-CoV-2 isolate from a COVID-19-positive patient’s nasal swab; from SYNLAB) was added to the wells containing sera dilutions, and the mixtures were incubated for 1 hour at 37°C. After this, 4x10^4^ Vero E6 cell suspension in 100 μl of VGM per well was added to the wells. Plates were incubated at 37°C and 5% CO_2_ for up to 5 days. Neutralization titer was calculated by microscopically evaluating cytopathic effect (CPE) in infected wells (round/floating cells compared to an untouched monolayer in non-infected wells). The dilution of sera that did not show CPE was considered a neutralization titer.

### CD4^+^ Memory T Cell Responses

PBMC were isolated from CPT tubes (BD Biosciences) according to the manufacturer’s instructions and cryopreserved in CTL-CryoTM ABC Media Kit (ImmunoSpot). Before T cell stimulation the PBMC vials were thawed, washed, counted, and suspended in an X-Vivo15 serum-free medium (Lonza). 1-2 million of PBMCs were used per condition. Overlapping peptide pools of SARS-CoV2 spike, nucleocapsid, and membrane proteins were purchased from Miltenyi Biotec and used at a final concentration of 1ug/ml of each peptide. The stimulations (mock, S, M, and N peptide pools) were carried out for 20 hours in the presence of anti-CD28 and anti-CD49d. CEFX peptide pool (JPT Peptides) was used as a positive control. After the stimulation T cells were stained with monoclonal antibodies: CD3 Brilliant Violet 650, CD4 Alexa Fluor 700, CD8 Brilliant Violet 605, CCR7 PE-Dazzle, CD45RA APC, CD69 Brilliant Violet 510 (all from Biolegend), and CD137 PE (from Miltenyi Biotech). Before the acquisition with LSRFortessa (BD Biosciences), 7AAD was added for the discrimination of dead cells. Data were analyzed using FCS Express 7 (DeNovo Software).

### Olink Proximity Extension Profiling

A panel of 92 inflammation-related biomarkers was measured in serum by the Proximity Extension Assay technique by Olink Proteomics. The assay uses two oligonucleotide-conjugated antibodies that bind to protein targets. Upon binding to the protein epitope, the paired oligonucleotide sequences are amplified through a quantitative real-time PCR reaction. Data is then generated using normalized protein expression (NPX) values on a log2 scale whereby a higher NPX correlates with higher protein expression. Proteins containing NPX values >50% below the assay’s limit of detection (LOD) were excluded from the analysis. The data were pre-processed by Olink^®^ using NPX Manager software.

### Statistics

The Wilcoxon Kruskal-Wallis tests with Dunn multiple correction and Spearman correlation calculations were performed using GraphPad version 9. The significance symbols on violin and dot plots are marked: * = adj. P ≤ 0.05, ** = adj. P ≤ 0.01, *** = adj. P ≤ 0.001 and **** = adj. P ≤ 0.0001.

## Results

### Decreased SARS-CoV-2 N but Stable S-RBD Antibodies in Asymptomatic Individuals

Using population-based serological screening we identified 56 SARS-CoV-2 antibody-positive individuals (20), from which 44 were seropositive without any sign of infection since March 2020 (asymptomatic) and 12 individuals who self-reported infection and were tested positive for the SARS-CoV-2 PCR test (symptomatic). We employed an automated CLIA and in-house LIPS method to detect SARS-CoV-2 antibodies. In the LIPS approach, we measured separately antibodies to N protein and S-RBD, the latter correlating with the antibody neutralization activity (23). Overall the CLIA method, targeting SARS-CoV-2 N protein, correlated well with LIPS N protein assay (r= 0.89, p<0.0001, **Supplementary Figure 1A**), slightly less with LIPS S-RBD (r= 0.59, p=0.0001, **Supplementary Figure 1B**), whereas there was a strong correlation between LIPS S-RBD and N findings (r=0.72, p<0.0001, **Supplementary Figure 1C**) among positive samples.

**Figure 1:**
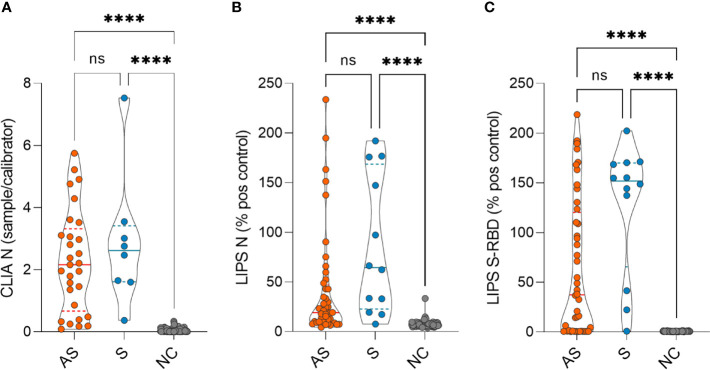
SARS-CoV-2 antibody levels in asymptomatic (AS), symptomatic (S), and negative control (NC) individuals as measured by **(A)** CLIA (n= 29 AS, 8 S and 115 NC) and **(B)** LIPS to N protein (n= 44 AS, 12 S and 115 NC) and **(C)** LIPS to S-RBD (n= 44 AS, 12 S and 115 NC) 7-8 months after the infection. CLIA is given as a fold difference of sample *vs* calibrator and LIPS results as the % of the positive control. Statistical differences were calculated with Kruskal-Wallis test with Dunn's multiple comparison testing. The statistical significance on violin plots is marked as **** = adj. P ≤ 0.0001; ns, non-significant.

**Figure 2:**
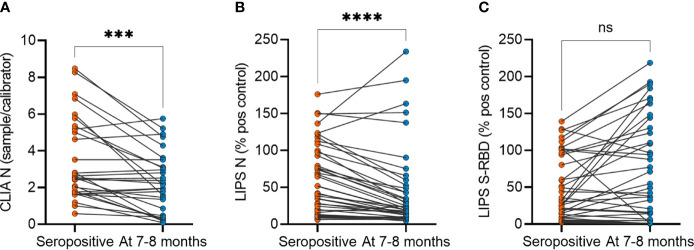
SARS-CoV-2 antibody levels in asymptomatic individuals at seropositivity and at 7-8 months after the infection. **(A)** CLIA (n= 29), **(B)** LIPS to N protein (n=44) and **(C)** LIPS to S-RBD (n=44). CLIA is given as a fold difference of sample *vs* calibrator and LIPS results as the percent of the positive control. Statistical differences were calculated with Wilcoxon two-tailed test. The statistical significance on dot plots is marked as *** = adj. P ≤ 0.001 and **** = adj. P ≤ 0.0001; ns, non-significant.

The antibody levels between asymptomatic and symptomatic patients were not different at 7-8 months but were significantly higher compared to negative controls as measured by CLIA (**Figure 1A**) and both LIPS assays (**Figures 1B, C** **)**. As the humoral response in SARS-CoV-2 infected individuals has been reported to decline with time, we studied the persistence of SARS-CoV-2 antibody levels at 7-8 months compared to the time point of seropositivity finding. Indeed, we found a significant decrease in SARS-CoV-2 N protein antibody levels in asymptomatic individuals by CLIA (**Figure 2A**) and LIPS N (**Figure 2B**) assays as well as in symptomatic individuals by CLIA (**Supplementary Figure 2**). In contrast, the antibody levels to S-RBD were not significantly changed (**Figure 2C**). Specifically, antibodies to N protein decreased in 82% (36 out of 44), whereas antibodies to S-RBD were lower in 54% (24 out of 44) of asymptomatic individuals. Moreover, S-RBD antibodies at 7-8 months correlated with the virus neutralization titers (r=0.421; p<0.005) (**Supplementary Figure 3A**), and similarly to S-RBD antibody levels, the neutralization titers were stable without significant change 7-8 months after the infections (**Supplementary Figure 3B**). The antibody levels among the sexes and age groups were similar. These results show that antibody levels to SARS-CoV-2 remain slightly decreased or stable 7-8 months after the infection. Although the antibodies to N protein decline with time, the S-RBD antibodies remain stable and, importantly, correlate with the virus neutralization capacity.

### T Cell Responses in Asymptomatic Individuals

**Figure 3:**
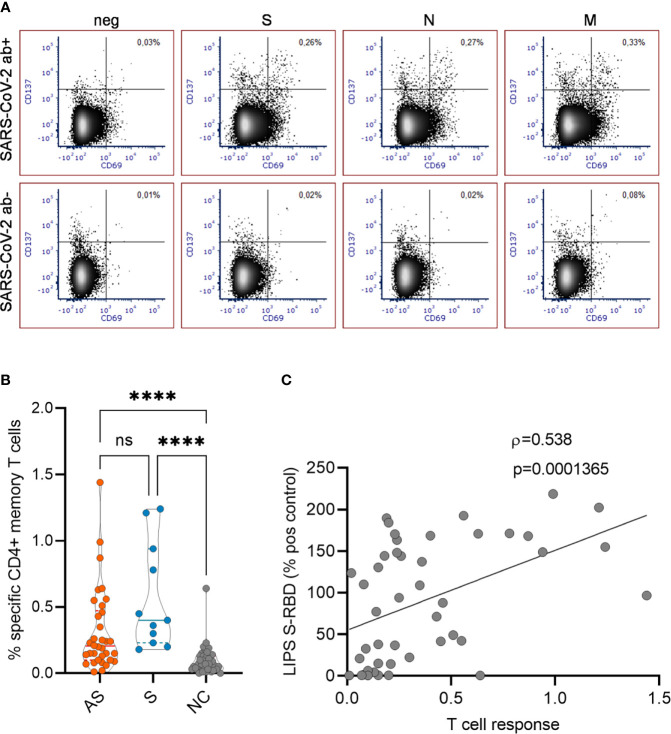
**(A)** Representative FACS plots showing SARS-CoV-2 specific CD4^+^ T cell responses in antibody positive and negative individuals. The proportion of CD137+ CD69+ activated CD4^+^ T cells with no peptides or with SARS-CoV-2 S, N and M peptide pools are given in the upper right quadrant. **(B)** The percentages of activated CD4^+^ T cells in asymptomatic (AS, n=34), symptomatic (S, n=11), and negative control (NC, n=41) individuals. **(C)** Correlation of specific T cell responses with antibody levels to S-RBD (n=45). Kruskal Wallis test with Dunn’s multiple comparison testing was used to calculate differences in **(B)** and Spearman’s rank correlation analysis was used in **(C)**. The statistical significance on violin plot is marked as **** = adj. P ≤ 0.0001; ns, non-significant.

We next analyzed the SARS-CoV-2 -specific memory T cell responses in asymptomatic and symptomatic antibody-positive individuals 7-8 months after their infection time. To this end, we tested the antigen-specific CD4^+^ T cell activation based on the upregulation of CD137 and CD69 activation markers on memory cells (single- and double-negative cells for CCR7^+^ and CD45RA^-^) using three SARS-CoV-2 antigen peptide mixes of S, N, and M proteins (**Figure 3A**) in 45 antibody-positive (34 asymptomatic and 11 symptomatic) and 41 antibody-negative individuals. The CD4^+^ T cells responded the strongest to M (mean % of activated CD4^+^ memory T cells 0.11; range 0%-0.47%) and S (0.10%; 0%-0.40%) derived peptides and had a slightly weaker response to N peptides (0.096%; 0%-0.82%). Calculating the sum of the three antigen responses among memory T cells showed a significantly higher proportion of antigen-specific activated CD4^+^ T cells among asymptomatic antibody-positive individuals (0.31%; 0%-1.44%) compared to negative controls (0.08%; 0%-0.23%) (**Figure 3B**), and though the symptomatic group (0.57%; 0.20%-1.24%) tended to have a higher number of positive T cells, this was not significant when compared to asymptomatic group. Further correlation with corresponding SARS-CoV-2 antibody levels showed CD4^+^ T cell responses to have a moderate but significant correlation (Spearman ρ= 0.54, p<0.001) with anti-RBD antibodies (**Figure 3C**) but not with anti-N antibodies as tested by CLIA and LIPS assays.

### Elevated Inflammation Serum Protein Levels in Asymptomatic Individuals

**Figure 4:**
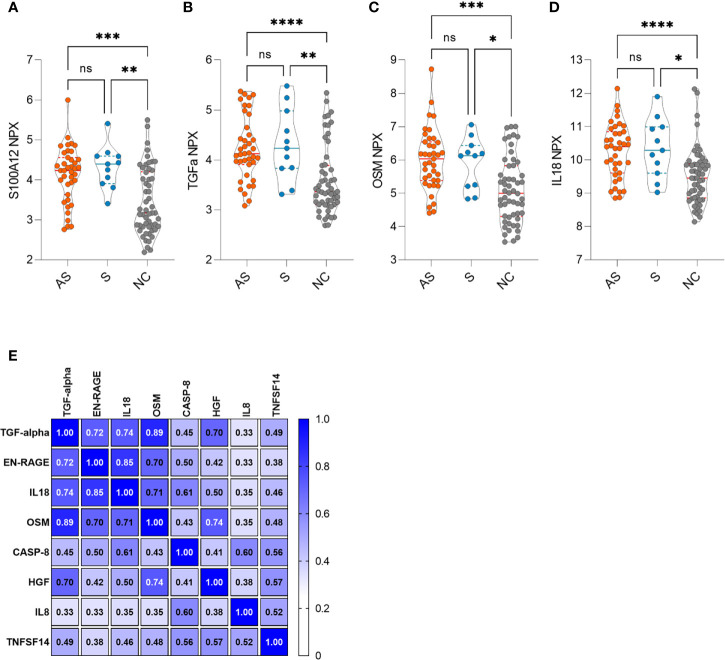
**(A–D)** Increased inflammation markers in asymptomatic (AS, n=39), symptomatic (S, n=11) and negative control (NC, n=54) individuals. **(E)** Clustered correlation matrix of eight inflammation markers. The statistical differences in violin plots were calculated with Kruskal-Wallis test with Dunn's multiple comparison testing. The statistical significance on violin plots is marked as * = adj. P ≤ 0.05, ** = adj. P ≤ 0.01, *** = adj. P ≤ 0.001 and **** = adj. P ≤ 0.0001; ns, non-significant. The Spearman correlation r values are given in the correlation matrix.

In search of signs of long-term inflammation in asymptomatic and symptomatic individuals, we applied Olink technology and profiled their serum samples for inflammation proteins. The screening of 50 antibody-positive (39 asymptomatic and 11 symptomatic) and 54 antibody-negative individuals showed overall similar levels in the majority of markers 7-8 months after the infection. Nevertheless, four serum proteins, S100A12, TGF-alpha, IL18, and OSM, all associated with activated macrophage-monocytic cells, were elevated in both the asymptomatic and symptomatic groups (**Figures 4A–D**). The four markers were thus more likely associated with the long-term effect of SARS-CoV-2 infection and not with symptoms of the disease. Within this panel, we searched for other inflammation markers associated with the four markers and found four other protein biomarkers (CASP8, HGF, TNFSF14, and IL8) which were in moderate correlation with elevated markers identified in seropositive individuals (**Figure 4E**). We did not see the elevated inflammation markers to correlate with antibody levels, T cell responses, or sex and age.

## Discussion

We here studied antibody and T cell responses to the SARS-CoV-2 virus and analyzed inflammation markers in asymptomatic individuals 7-8 months after the infection. We found a decrease in antibodies to N protein but relatively stable levels of S-RBD antibodies. Asymptomatic individuals developed memory T cell responses, which correlated with S-RBD antibodies, and a small subset of them had persistently elevated levels of monocyte-macrophage-associated inflammatory protein levels in their blood.

We here found decreased antibodies to SARS-CoV-2 N protein over time, which we detected by commercial CLIA and in-house LIPS assay, however, the S-RBD antibody levels were not changed. Importantly, the S-RBD antibodies correlated with the virus neutralization and with T cell responses to SARS-CoV-2 N, S, and M proteins. Earlier studies in SARS-CoV-2 infected asymptomatic individuals and in patients with mild disease have mostly reported the virus-specific antibodies to decline over time, or stable in some studies. In a study with more than 200 individuals, S-RBD IgG titers were found relatively stable, though the moderate decline in antibodies was seen in over 8 months (7). In another longitudinal study including 1965 healthcare workers with no or mild symptoms, Havervall and colleagues reported stable antibodies to the SARS-CoV-2 S protein in 96% and N protein in 80% of individuals at four months post-infection (24). Other studies have reported prominent decline in antibodies to S protein. Long et al. found 93% of asymptomatic individuals to have a reduction in anti-SARS-CoV-2 antibody levels during the early convalescent phase (5). Chen et al. found a significant decline in anti-S-RBD antibodies 7 months after the infection in hospitalized cases (25). The decline in antibody levels to N and S-RBD after 5 months was also reported by Gaebler et al. (26). We note that in the asymptomatic group of our study, the S-RBD antibody levels showed more individual pattern as they increased in some and decreased in others. Our results also suggest that testing for both S-RBD and N protein antibodies should be considered to evaluate the long-term antibody responses in infected individuals.

We here report an unexpected finding of higher levels of inflammation markers associated with monocyte-macrophage lineage. S100A12, TGF-alpha, IL18, and OSM were higher in antibody-positive individuals after 7-8 months suggesting their involvement in SARS-CoV-2 pathogenesis. S100A12 is known as a lymphocyte-activating alarmin, expressed by activated monocyte-macrophages and neutrophils (27). The upregulated serum levels of S100A12 and OSM are characteristic for patients with severe COVID-19 (23, 28), but as they are overexpressed at sites of inflammation, their serum levels correlate with the disease activity of many inflammatory diseases, including sepsis and other pulmonary infections (29, 30). Thus, our results strongly suggest a persistent pulmonary inflammation process even in patients with asymptomatic or mild SARS-CoV-2 infection. The inflammation process may be associated with prevalent radiological findings of lung opacities and airway abnormalities in a third of the asymptomatic cases (3) but its long-term persistence months after the infection is unexpected.

Our study has limitations. First, although our cohort included sex- and age-matched individuals from the same geographical regions, we cannot exclude the possibility that other comorbidities or infections may influence the inflammatory protein levels in studied individuals. Second, we have not studied the role of seasonal coronaviruses, which may affect our results on T cell responses or antibody levels. Third, we did not study the antigen-specific memory B cells to confirm the persistence of B cell responses to SARS-CoV-2. Further studies are needed to identify whether the elevated levels of inflammation markers are related to the long-term effect of the viral pulmonary inflammation or pose a risk factor for the increased infectivity for the SARS-CoV-2 virus.

## Data Availability Statement

The raw data supporting the conclusions of this article will be made available by the authors, without undue reservation.

## Ethics Statement

The studies involving human participants were reviewed and approved by The Ethical Committee at the University of Tartu. Written informed consent to participate in this study was provided by the participants’ legal guardian/next of kin.

## Author Contributions

LT, JM, AH, and EZ did the experiments. DI and MS collected the biological samples of studied individuals. PJ, PN, KKa, KKi, IL, and PP contributed to conception and design of the study. FL and KKa organized the database. AS, HS, and KT performed the statistical analysis. PP wrote the manuscript. PJ, PN, EZ, KKi, and IL wrote sections of the manuscript. All authors contributed to the article and approved the submitted version.

## Funding

The study was supported by the Centre of Excellence in Translational Genomics (EXCEGEN), and the Estonian Research Council grants PRG377 and PRG1117. The study was funded by the Ministry of Social Affairs of the Republic of Estonia. Electronic data collection was supported by the High Performance Computing Center of the University of Tartu.

## Conflict of Interest

Author PN was employed by company SYNLAB Estonia.

The remaining authors declare that the research was conducted in the absence of any commercial or financial relationships that could be construed as a potential conflict of interest.

## Publisher’s Note

All claims expressed in this article are solely those of the authors and do not necessarily represent those of their affiliated organizations, or those of the publisher, the editors and the reviewers. Any product that may be evaluated in this article, or claim that may be made by its manufacturer, is not guaranteed or endorsed by the publisher.

## Supplementary Material

Supplementary Figure 1 Correlation between the antibody analysis methods CLIA, LIPS-N, and S-RBD (n=36). Correlation coefficients were calculated with Pearson correlation analysis.

Supplementary Figure 2 SARS-CoV-2 antibody levels in symptomatic individuals (n=12) at seropositivity and 7-8 months after the infection. **(A)** CLIA and **(B)** LIPS to N protein and **(C)** LIPS to S-RBD. CLIA is given as a fold difference of sample *vs* calibrator and LIPS results as the percent of the positive control. Statistical differences are calculated with Wilcoxon two-tailed test.

Supplementary Figure 3 **(A)**. Correlation analysis between the sample neutralization and LIPS S-RBD antibody values (n=42). Spearman’s rank correlation analysis was used. **(B)** Neutralization titers at seropositivity analysis and 7-8 months after the infections (n=42).
