# Supplementary figures and images for: Long-Term Elevated Inflammatory Protein Levels in Asymptomatic SARS-CoV-2 Infected Individuals
Source: Front Immunol. 2021 Sep 17;12:709759. doi: 10.3389/fimmu.2021.709759 (PMC8484961; doi:10.3389/fimmu.2021.709759)

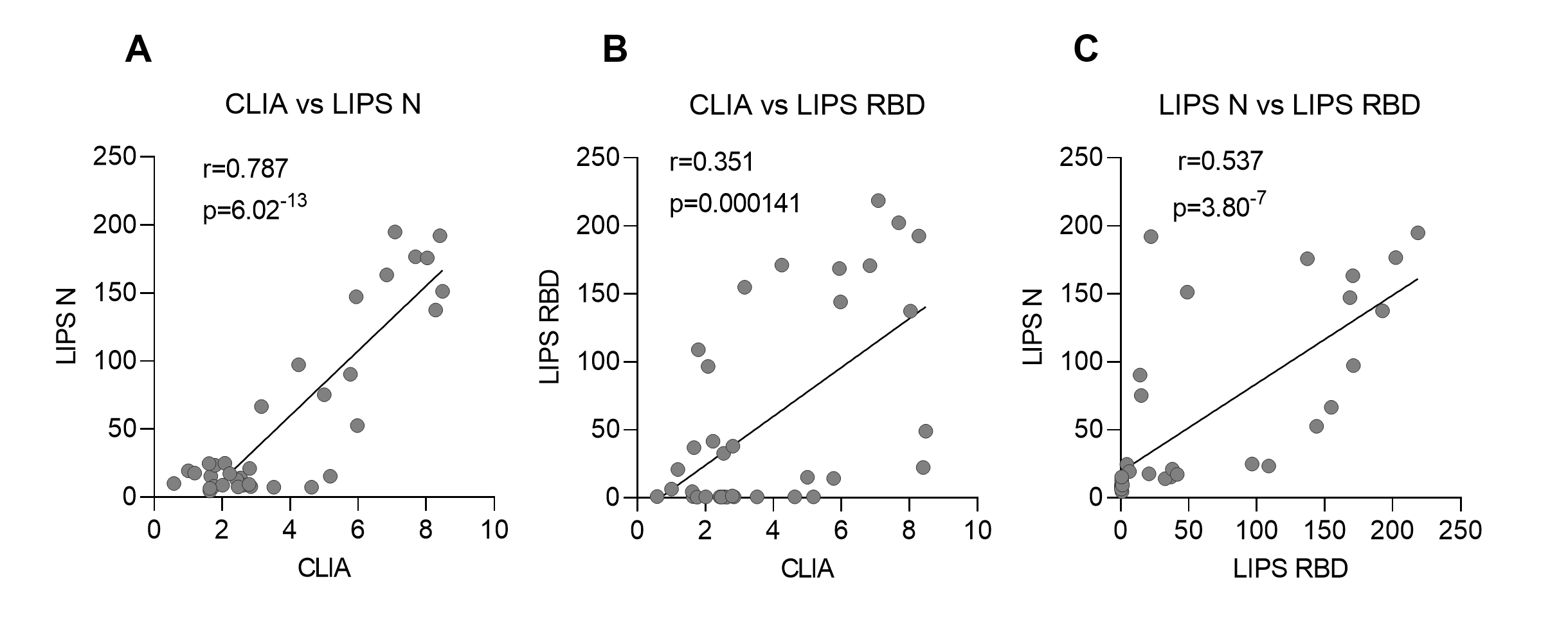

Supplement: Supplementary Figure 1 — Correlation between the antibody analysis methods CLIA, LIPS-N, and S-RBD (n=36). Correlation coefficients were calculated with Pearson correlation analysis. [file Image_1.tif]

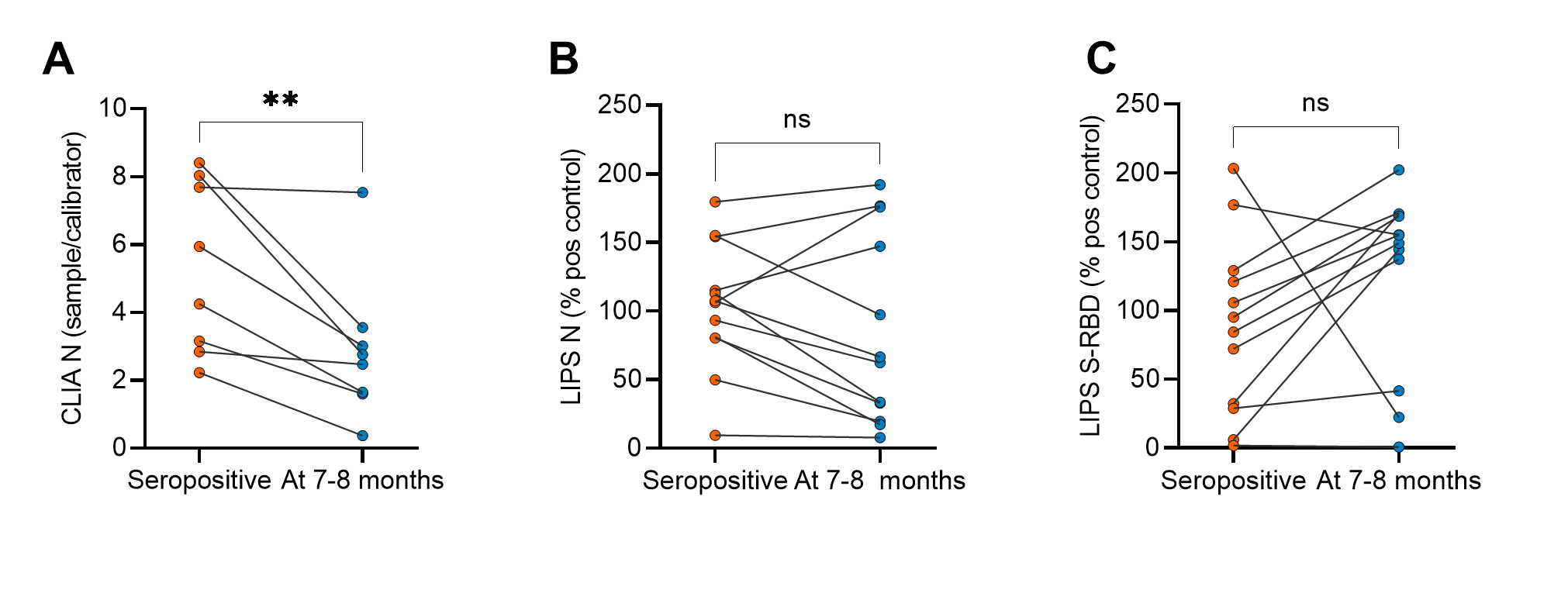

Supplement: Supplementary Figure 2 — SARS-CoV-2 antibody levels in symptomatic individuals (n=12) at seropositivity and 7-8 months after the infection. (A) CLIA and (B) LIPS to N protein and (C) LIPS to S-RBD. CLIA is given as a fold difference of sample vs calibrator and LIPS results as the percent of the positive control. Statistical differences are calculated with Wilcoxon two-tailed test. [file Image_2.tif]

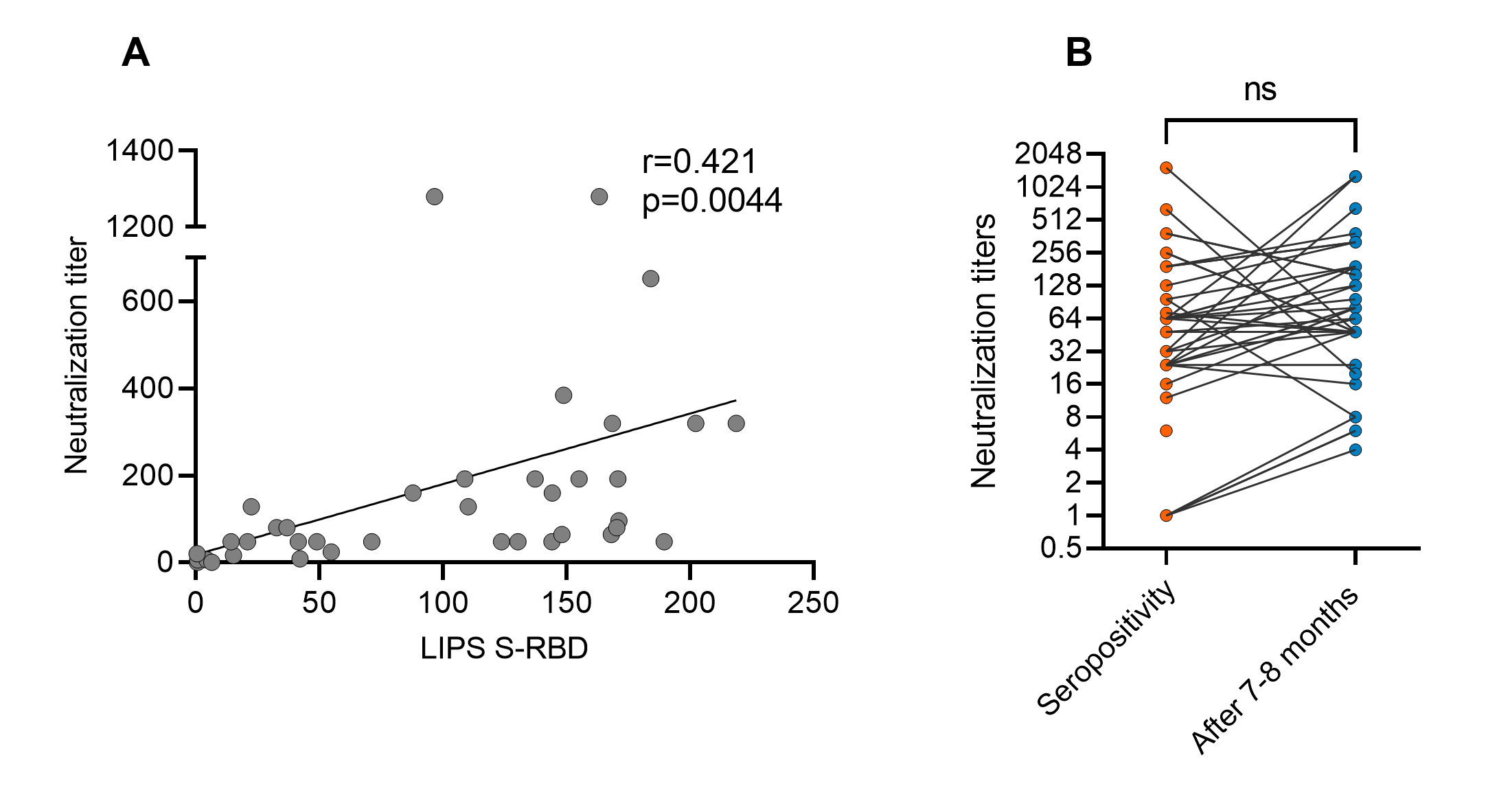

Supplement: Supplementary Figure 3 — (A). Correlation analysis between the sample neutralization and LIPS S-RBD antibody values (n=42). Spearman’s rank correlation analysis was used. (B) Neutralization titers at seropositivity analysis and 7-8 months after the infections (n=42). [file Image_3.tif]
